# A Systematic Review of Donor Serum Sodium Level and Its Impact on Transplant Recipients

**Published:** 2020

**Authors:** J. Basmaji, L. Hornby, B. Rochwerg, P. Luke, I. M. Ball

**Affiliations:** 1 *Division of Critical Care, Department of Medicine, Schulich School of Medicine and Dentistry, Western University, London, Ontario, Canada*; 2 *Children’s Hospital of Eastern Ontario Research Institute, Ottawa, Ontario, Canada*; 3 *Canadian Blood Services, Ottawa, Ontario, Canada*; 4 *Division of Critical Care, Department of Medicine, McMaster University, Hamilton, Ontario, Canada*; 5 *Department of Health Research Methods, Evidence, and Impact, McMaster University, Hamilton, Ontario, Canada*; 6 *Division of Urology, Department of Surgery, Western University, London, ON, Canada*; 7 *Matthew Mailing Centre for Translational Transplant Studies, Lawson Research Institute, London Health Sciences Centre, London, Ontario, Canada*

**Keywords:** Transplantation, Organ donation, Sodium, Brain death

## Abstract

**Background:**

An important aspect of donor management is the optimization of serum sodium levels.

**Objective:**

To perform a systematic review to determine the effects of donor sodium levels on heart, lung, kidney, and pancreas graft function, recipient mortality, and to identify the optimal donor serum sodium target.

**Methods:**

We searched MEDLINE, Cochrane, Guideline databases, and trial registries from 1946 to May 2019 for studies investigating the effects of donor serum sodium levels on transplant outcomes in all non-hepatic organs. A two-step independent review process was used to identify relevant articles based on inclusion/exclusion criteria. We describe the results narratively, assess the risk of bias, and apply GRADE methodology to evaluate the certainty in the evidence.

**Results:**

We included 18 cohort studies in our final analysis (n=28,007). 3 of 4 studies demonstrated an association between donor serum sodium and successful organ transplantation. 5 studies reported no association with graft function, while 6 studies did. 5 studies reported on recipient survival, 3 of which suggested donor sodium is unlikely to be associated with recipient survival. The included studies had serious risk of bias, and the certainty in evidence was deemed to be very low.

**Conclusion:**

In low risk of bias studies, donor sodium dysregulation is unlikely to affect kidney graft function or mortality of heart and kidney recipients, but the certainty in the evidence is very low due to inconsistency and imprecision. Further research is required to refine the serum sodium target range, quantify the dose-response curve, and identify organs most vulnerable to sodium dysregulation.

## INTRODUCTION

Despite advances in deceased donor management in recent decades, the demand for organs exceeds supply [1]. In light of this, strategies to optimize donor care to improve the quality and quantity of organs available are a key priority for national donation and transplant organizations [2]. One important aspect of donor management is the optimization of serum sodium levels. 

Both hypo- and hypernatremia have been linked to adverse effects in transplant recipients. Hyponatremia is common in patients with acute kidney injury (AKI) of pre-renal origin and may be a marker of progressive AKI in donor kidneys [3]. Disease processes such as cerebral salt wasting (CSW) can also precipitate hyponatremia and hypovolemia, leading to compromised organ perfusion. Conversely, hypernatremia has been linked to excessive sodium infusions during prolonged ICU stays, portending a high burden of critical illness [4]. It is also associated with the development of diabetes insipidus after brain death, a process that leads to progressive hypovolemia and compromised organ perfusion [5].

Whether sodium dysregulation adversely affects graft outcomes independently or whether it is a surrogate for other processes has not yet been elucidated and generates considerable discussion in the literature [6]. Elevated intracellular sodium levels due to hypernatremia can lead to cellular swelling and tissue dysfunction, further exacerbated by ischemic reperfusion injuries [7, 8]. While the detrimental effects of sodium dysregulation are well known at a cellular level, its effect on patient-centered outcomes has not been consistently demonstrated. Some studies have shown harm associated with donor dysnatremia [9, 10], while other studies have not [11, 12]. To date, the majority of studies examining the effect of donor sodium levels on recipient outcomes has been in the context of liver transplantation, with less attention given to other solid organs [13-17]. In this article, we present a systematic review of the literature and summarize the available evidence regarding the association between maintenance of donor sodium in the normal range compared with permissive hyper- or hyponatremia and transplant outcomes for heart, lung, kidney, and pancreas recipients.

## MATERIALS AND METHODS

Our systematic review followed the Cochrane Handbook for Systematic Reviews and Interventions, and reported results according to the PRISMA guideline [18].

Search Question, Population, Eligibility Criteria

The objective of our review was to determine whether maintenance of sodium in the normal range, compared with hypo- or hypernatremia, improves the organs transplanted per donor, recipient graft function and graft survival, and recipient mortality. For recipient graft function, survival, and recipient mortality, we focused on studies reporting outcomes in heart, lung, kidney, and pancreas recipients. We included studies of pediatric or adult potential organ donors declared deceased by neurological criteria. We defined the normal sodium range as 135–145 mEq/L. Since we anticipated identifying only observational studies, cohorts with mean or median sodium values outside of the normal range were included. We examined the following outcomes: clinical status during the interval between death declaration and organ recovery (temperature, hemodynamics, oxygenation, and metabolism), organ acceptance/recovery/transplantation, recipient quality of life, graft function, graft survival, and recipient survival, without a pre-specified follow-up duration. 

We included all prospective and retrospective studies, but excluded scoping reviews, systematic reviews, and meta-analyses. We excluded experimental/animal studies, non-research articles, and conference abstracts. Articles not published in English or French were also excluded.

Search Strategy

We performed unrestricted searches in MEDLINE and Cochrane up until May 2019 to identify relevant articles. Trial registry records and clinical practice guidelines were also searched to identify relevant studies. With input from the study investigators, a health information specialist performed the search with appropriate wildcards to consider plurals and variations in spelling. Relevant articles were then reviewed by the health information specialist to confirm the validity of the search strategy and for emerging themes not already accounted for. The search strategy was revised accordingly and the search was repeated. The full search strategy is available in the Appendix. The search included articles published in English and French. Reference lists of relevant articles were manually searched to identify additional relevant articles.

Study Selection and Data Extraction

Articles were screened over two stages. In the first stage, two reviewers (LH and PL) independently reviewed the titles and abstracts, and selected relevant articles for full text review. A third reviewer (JB or IB) resolved disagreements. Two reviewers (JB and LH) independently reviewed the full texts of the articles selected in the first stage. Disagreements between reviewers were discussed in conjunction with a third reviewer (IB) in order to reach consensus.

Two reviewers (JB and LH) independently extracted data in duplicate through a standardized form. We collected study design, number of patients, donor characteristics, sodium levels, and relevant outcome data. The impact of various donor sodium thresholds on outcomes were summarized primarily by treatment-effect ratios. We contacted the corresponding authors for data points of interest that were not reported in the studies. Disagreements were resolved by discussion first, followed by arbitration by a third party (IB), if required. 

Evaluating the Risk of Bias and Grading of the Evidence

We used the Newcastle Ottawa Scale (NOS) to assess the risk of bias across studies [19]. Based on the NOS, studies with 7–9 stars were considered “high quality” studies with low risk of bias; studies with 4–6 stars were considered “fair quality” studies with moderate risk of bias; and studies with 3 stars or less were considered “low quality” studies with serious risk of bias. Since hemodynamic status can confound the effect of donor sodium on transplant outcomes, the comparability domain required controlling for vasopressor requirements (including doses) and volume status.

We evaluated the certainty in evidence using the Grading of Recommendations Assessment, Development, and Evaluation (GRADE) system [20]. In keeping with GRADE guidance, observational studies started as low quality evidence. From there, we downgraded certainty in evidence at an outcome level based on assessments of the following domains: risk of bias, imprecision, indirectness, and inconsistency. Given the heterogeneity of the studies, we were unable to perform a meta-analysis. 

## Results

**Figure 1:**
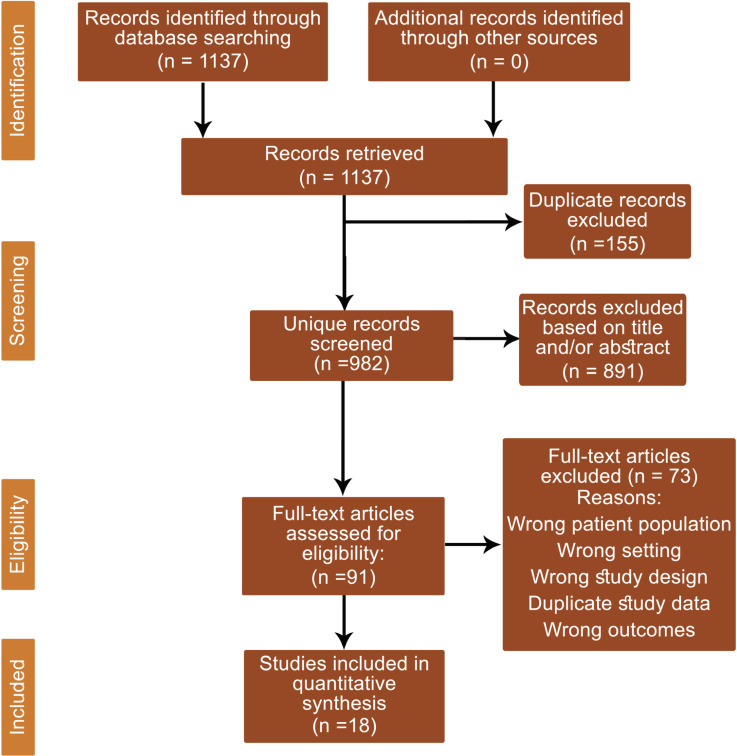
PRISMA diagram showing systematic selection of records for review

Of the initial 1137 citations identified in the search, 982 unique records were screened. From the screened records, 91 full-text articles were reviewed, and 18 studies met our inclusion criteria (Fig 1). The majority of excluded studies were conference abstracts or narrative reviews with no original research. Other major reasons for exclusion included incorrect patient population, citations not relevant to the study question, a reporting of transplanted graft outcomes without reporting on donor sodium levels, or studies that utilized the same patient cohort.

Organs Transplanted Per Donor

Four studies reported on donor sodium levels’ association with successful organ transplantation [21-24]. Three of the four studies reported the outcome as the probability of successful transplantation [21-23], while one study investigated factors predictive of successful pancreatic transplantation [24]. An overview of the studies is provided in Table 1.

**Table 1:** Observational studies evaluating donor serum sodium and associated clinical outcomes

Study	Cohort Size	Organ	Donor Serum Na^+^ Target (mEq/L)	Outcome	Findings
Organs Transplanted Per Donor					
Drewitz *et al*, 2014	3666	Pancreas	≥160	Transplantation of pancreas	Independent predictor of pancreas non-transplantation
Franklin *et al*, 2010	467	-	≥160	Probability of organ transplanted	Increased probability of heart transplantation. No effect on probability of transplantation of other organs
Malinoski *et al*, 2011	320	-	135–160	Organs transplanted per donor (OTPD)	Achieving donor serum Na^+^ of 135–160 mEq/L predicted ³4 OTPD
Selck *et al*, 2008	14125	-	146.41±9.23	OTPD	No effect on organs transplanted per donor
Graft Function					
Baptista *et al*, 2013	1298	Kidney	154.6±15.4	Delayed graft function	No association with graft function
Dominguez *et al*, 2009	212	Kidney	149.9±13.2	Delayed graft function	No effect
Finfer *et al*, 1996	70	Kidney	163.4±10.9	Primary non-functionEarly graft function	Not independent predictor of graft dysfunction
Gallinat *et al*, 2016	78	Kidney	149 (129–185)	Delayed graft function	No effect
Jung *et al*, 2010	74	Kidney	151.1±10.9	Delayed graft function	Not a predictor of kidney graft function
Kazemeyni *et al*, 2008	57	Kidney	>155	Creatinine at follow-up	Donor hypernatremia correlated with higher creatinine values
Kwiatkowksa *et al*, 2017	89	Kidney	>155	Early graft functionLate graft function	No increased risk of early graft function Increased risk of late graft function
Park *et al*, 2018	271	Kidney	162.4±11.1	Delayed graft function	Independent predictor of delayed graft function
Rangel *et al*, 2010	150	Kidney-Pancreas	>155	Delayed graft function	Independent predictor of delayed kidney graft function
Stolyar *et al*, 2015	342	Kidney	149±12	Graft survival	Associated with reduced graft survival
Tian *et al*, 2008	42	Kidney	152.4±13.4	Delayed graft function	Mean sodium values were not different between grafts with normal function and dysfunctional grafts
Recipient Survival					
Chen *et al*, 2002	305	Heart	Not specified	45-day mortality 1-year mortality	No effect on recipient mortality
Gallinat *et al*, 2016	78	Kidney	149 (129–185)	5-year survival	No effect on recipient survival
Hoefer *et al*, 2010	4641	Heart	130–169	1-year mortality	Increased recipient mortality
Kaczmarek *et al*, 2006	1800	Heart	Quartiles set at 141, 147, and 154	30 day, 5- and 10-year mortality rates	No effect on recipient mortality
Stolyar *et al*, 2015	342	Kidney	149±12.1	Recipient survival^b^	Increased recipient mortality

^a^ PDF was defined as initial poor function (AST >2500 U/L or clotting factor support >2 d) or primary non-function (re-transplantation required within 7 days)

^b^ End of observation was up to 162 months.

In Malinoski, *et al*’s retrospective study on donor management goals (DMGs), achieving a sodium maintenance in the range of 135–160 mEq/L was an independent predictor of four or more organs transplanted per donor (OR: 3.35, 95% CI 1.14–9.95) [21]. Interestingly, the mean±SD values of sodium between those who successfully donated four or more organs versus those that did not was not significantly different (146.3±11.2 mEq/L *vs* 147.3±10.2 mEq/L, p=0.418). Donors with four or more organs transplanted were more likely to have achieved this target (50.4% *vs* 31.4%, p=0.035).

Franklin, *et al*, investigated the effects of DMGs on transplantation success [22]. Their study took place over a two-phase period where DMGs were implemented and modified based on interim data analysis. In the first cohort of 338 donors, achieving a sodium of ≤155 mEq/L did not increase the probability of successful organ transplantation. In the second phase, a sodium of <160 mEq/L had a negative effect on the rate of cardiac transplantation (OR 0.4, 95% CI 0.2–0.8).

Selck, *et al*, retrospectively reviewed the United Network for Organ Sharing (UNOS) registry to identify variables predictive of the number of organs transplanted per donor (OTPD) [23]. In their cohort of 14,125 patients, the final donor sodium level was not found to predict OTPD (ordinary least squares regression, p=0.564). Desmopressin administration and diuretic usage were both predictive of OTPD (p<0.001 and p=0.001, respectively), potentially accounting for the association between donor sodium and the quantity of organs procured.

We identified only one study investigating the effect of donor sodium on pancreatic transplantation. Drewitz, *et al*, retrospectively, reviewed the Eurotransplant database to identify factors predictive of pancreas non-transplantation [24]. Donor sodium levels ≥160 mEq/L independently predicted pancreas non-transplantation (OR 1.75, 95% CI 1.18–2.61).

Kidney Graft Function and Survival 

We identified 11 studies that analyzed the effect of donor dysnatremia on renal graft function and survival. Of those, five studies found an association between donor hypernatremia and recipient graft function [10, 25-28], while six did not [12, 29-33]. An overview of the studies is provided in Table 1.

Of the five studies concluding an association between donor sodium and kidney graft function, three defined the exposure as a sodium value >155 mEq/L. In the retrospective cohort of 89 kidney recipients by Kwiatkosa, *et al*, sodium concentrations >155 mEq/L were associated with a significantly increased risk of graft dysfunction over the 5-year follow-up period (log rank test, p=0.036) and the hazard ratio for loss of graft function was 1.09 (95% CI 1.01–1.18) [26]. Long-term graft dysfunction was defined by creatinine levels and estimated creatinine clearance. There was no association between hypernatremia and early graft dysfunction, defined as the need for dialysis within 7 days post-transplant (p value was not reported). Kazemeyni, *et al*, arrived at a similar conclusion; in their cohort of 57 kidney transplant recipients, grafts from hypernatremic donors ([Na^+^] >155 mEq/L) had significantly higher creatinine values in follow-up (141.4 mmol/L vs 114.9 mmol/L, p=0.02) [27]. Donor serum sodium correlated with recipient creatinine (r=0.316, p=0.02). The median follow-up for patients was 20 (range: 2–36) months. For Rangel, *et al*’s cohort of 150 simultaneous kidney-pancreas transplants, their multivariate analysis identified donor serum sodium levels >155 mEq/L as a predictor of delayed graft function, defined as the need for dialysis in the first week post-transplantation (OR 1.09, 95% CI 1.04–1.15, p<0.0001) [28]. Park, *et al*’s cohort of kidney transplants had grafts retrieved from donors with extreme hypernatremia (161.7±11.7 mEq/L) [10]. The maximum sodium level was higher in the group with delayed graft function and hypernatremia was identified as an independent risk factor for delayed graft function in their multivariate analysis (OR 1.23, 95% CI 1.08–1.39, p=0.001). Only one study identified hyponatremia as an independent predictor of graft survival in their multivariate analysis over a 162-month follow-up period (Kendall k = 0.116; p=0.048) [25].

Six studies did not find an association between donor sodium and graft function. In two studies, the cohorts had a central tendency of donor sodium levels between 145 and 150 mEq/L (149.9±13.2 and 149 [range 129–185] mEq/L) and there was no association with delayed kidney graft function, defined as the need for dialysis in the first week post transplantation [32, 33].

Three studies reporting on donors with more significant hypernatremia (mean cohort values between 150 and 155 mEq/L) also found no association on graft function. Baptista, *et al*’s cohort found no difference between donor sodium levels in recipients with and without delayed graft function (155.3±15.3 mEq/L and 153.6±15.6 mEq/L, respectively, p=0.085) [12]. Hypernatremia was not predictive of delayed graft function (OR 1.01, 95% CI 1.0–1.0) or poor kidney function (OR 0.99, 95% CI 1.0–1.0). Delayed graft function was defined as the need for dialysis in the first 7 days post-transplant and poor kidney function was defined as a creatinine clearance less than 50 mL/min/1.73 m^2^. In Tian, *et al*’s cohort, donor mean sodium values did not differ between recipients with delayed graft function and those with normal post-transplant function (152.4±13.4 mEq/L *vs* 148.7±9.3 mEq/L, p=0.489) and did not independently predict acute rejection (OR 0.78, 95% CI 0.6–1.03) [29]. Delayed graft function and acute rejection episodes were not explicitly defined and this raises concern for risk of bias with respect to outcome ascertainment. Jung, *et al*’s study of 74 recipients also did not identify donor sodium as an independent risk factor for delayed graft function in a multivariate analysis (OR was not reported) [30].

We identified only one pediatric cohort in the literature, which also had the most extreme donor sodium mean (163.4±10.9 mEq/L). Despite this more extreme range of hypernatremia, there was no significant association between donor sodium levels and delayed graft function in recipients of pediatric kidney grafts (p=0.13) [31]. 

Recipient Mortality

Five studies described the effect of donor sodium on recipient survival for patients receiving heart, kidney, or kidney-pancreas grafts. Three of those studies conclude that sodium dysregulation has no effect on recipient mortality [11, 33, 34]; two of those studies found an association between sodium dysregulation and recipient mortality [9, 25]. An overview of the studies is provided in Table 1.

Of the two studies showing an association between donor sodium and recipient mortality, one evaluated heart donors and the other kidney donors. Hoefer, *et al*, retrospectively reviewed 4641 orthotopic heart transplants and applied a multivariate analysis to determine predictors of 1-year post-transplantation survival [9]. Recipients receiving a donor heart with serum sodium level <130 mEq/L or >170 mEq/L had an increased risk of 1-year post-transplantation mortality compared with donors with normal sodium levels (HR: 1.25, 95% CI 1.04–1.50). The 1-year post-transplantation survival was 74% in recipients with normal donor sodium ranges, and 64% in recipients with donor hypo- or hypernatremia (log rank test, p=0.007). Stolyar, *et al*, conclude that donor hyponatremia is an independent predictor of recipient mortality (Kendall k = 0.143; p=0.015) in their multivariate analysis over a 162-month follow-up period [25].

Of the four studies showing no association between donor sodium and recipient mortality, two pertained to heart donors, one to kidneys donors, and one to simultaneous kidney-pancreas transplantations. Kaczmarek, *et al*’s study regarding the effect of sodium levels on the survival of 1800 heart transplant recipients shows no association. Donors were divided into four groups (classified as A through D) based on their sodium levels. The quartiles used to divide donors were: 141, 147, and 154 mEq/L. For each group, 30-day mortality in group A was 9.3%; B, 10.0%; C, 10.0%; and D, 8.8% (p=0.22). Five- and 10-year survival rates in group A were 71.1% and 53.8%; B, 69.3% and 53.9%; C, 72.7% and 61.0%; and D, 71.2% and 62.4%; respectively (log-rank test, p>0.05) [11]. Chen, *et al*, investigated donor variables predictive of 45-day mortality and late 1-year all-cause mortality in heart transplant recipients [34]. Donor sodium (value was not specified) was not found to be predictive of recipient mortality (p value was not reported). Similarly, Gallinat, *et al*’s cohort also found no association between donor hypernatremia and kidney recipient survival at 5 years, respectively [33].  

Risk of Bias and GRADE Assessment of the Evidence

All studies were evaluated for risk of bias using the Newcastle Ottawa Scale. There were seven studies deemed to be high quality with low risk of bias (Table 2). Only one study found an association between donor sodium dysregulation and kidney graft function and survival (n=150), while three did not (n=1450). All high-quality studies with low risk of bias found no association between donor sodium dysregulation and transplant recipient mortality (n=1878). In one high quality study, donor dysnatremia only predicted unsuccessful heart transplantation, with no bearing on other organs (n=467). Another high-quality study found donor dysnatremia not predictive of successful organ transplantation (n=14,125). 

**Table 2:** Risk of bias summary of included studies

Certainty assessment						Impact	Certainty
No of studies	Study design	Risk of bias	Inconsistency	Indirectness	Imprecision		
Organs Successfully Transplanted							
4	Observational studies	Not serious	Serious^1^	Not serious	Serious^2^	Three studies showed an association between normalizing serum sodium and the number of organs transplanted per donor, while one study did not.	Very low
Kidney Graft Function							
11	Observational studies	Serious^3^	Serious^1^	Not serious	Serious^2^	Five studies showed an association between donor sodium dysregulation and recipient graft function, while six studies did not.	Very low
Recipient Survival							
5	Observational studies	Serious^3^	Serious^1^	Not serious	Serious^2^	Two studies showed sodium dysregulation had an effect on recipient mortality, while three studies did not.	Very low

^1^ Serious concerns about inconsistency were raised given the variable conclusions drawn regarding the effect of donor sodium on outcomes

^2^ Serious concerns about imprecision due to the confidence intervals suggesting both appreciable benefit and harm for specific donor sodium targets.

^3^ Serious concerns about representativeness of the cohort and ascertainment of exposure.

The certainty in evidence for organs successfully transplanted was very low due to concerns of inconsistency and imprecision. The certainty in evidence for graft function and survival, as well as recipient mortality, was downgraded to very low due to serious concerns with respect to risk of bias, imprecision, indirectness, and inconsistency. The variability in study design, patient populations, the exposure, and outcomes precluded a meta-analysis. The grading of the evidence is presented in Table 3.

**Table 3:** GRADE summary of findings for transplant outcomes

Study ID	Selection				Comparability	Outcome			Total Score
	Representativeness of exposed cohort	Selection of non-exposed cohort	Ascertainment of exposure	Outcome of interest not present at start of study	Comparability of cohorts on basis of design or analysis	Assessment of outcome	Length of follow-up	Adequacy of follow-up of cohorts	
Baptista *et al*, 2013	*	*		*	*	*	*	*	7
Chen *et al*, 2002		*		*		*	*	*	5
Dominguez *et al*, 2009		*		*		*		*	4
Drewitz *et al*, 2014	*	*	*			*	*	*	6
Finfer *et al*, 1996		*		*	*				3
Franklin *et al*, 2010	*	*	*		*	*	*	*	7
Gallinat *et al*, 2016		*		*	**	*	*	*	7
Hoefer *et al*, 2010		*		*	*	*	*	*	6
Jung *et al*, 2010	*	*		*	*	*	*	*	8
Kaczmarek *et al*, 2006	*	*	*	*	*	*	*	*	8
Kazemeyni *et al*, 2008		*	*	*					3
Kwiatkowska *et al*, 2017		*	*	*	*		*	*	6
Malinoski *et al*, 2011						*	*	*	3
Park *et al*, 2018		*		*	*	*	*		5
Rangel *et al*, 2010		*	*	*	**	*	*	*	8
Selck *et al*, 2008	*	*	*		**	*	*	*	8
Stolyar *et al, *2015		*		*		*	*	*	5
Tian *et al*, 2008		*		*			*	*	4

## DISCUSSION

Despite the increasing number of organ transplantations performed in recent years, many patients remain on organ transplant wait-lists, creating a sense of urgency to ensure all potential donors are converted to actual donors by optimizing donor care[2, 35]. Normalization of sodium dysregulation is widely regarded as an important aspect of liver donor management, but other solid organs have garnered less attention from the organ donation and transplant community. Our systematic review sought to address this knowledge gap by identifying the optimal donor sodium threshold for kidney, lung, pancreas, and heart transplant outcomes to better inform clinical practice guidelines in donor management.

There are two perspectives to the deleterious effects of sodium dysregulation in potential organ donors [11]. Sodium dysregulation may be a secondary manifestation of adverse processes such as diabetes insipidus, cerebral salt wasting, or excessive saline solution administration in hemodynamically unstable donors, or it can impose detrimental effects independent of these comorbid processes. In support of this hypothesis, hypernatremia has been implicated in reduced cardiac contractility, reduced peripheral vascular resistance, impaired lactate clearance, and impairment of endothelial and glycocalyx barrier function, free radical production, and ischemic reperfusion injury [36]. These theories have not consistently been demonstrated with regard to clinical outcomes.

Only two studies identified a relationship between donor hypernatremia and kidney graft function for donors with sodium levels >155 mEq/L. Conversely, the mean donor sodium levels in the cohorts of negative studies ranged from 149 to 154.6 mEq/L. The sodium levels in these cohorts may not have been extreme enough to bring about graft dysfunction, raising the question of a dose-response relationship that should be an area of further study. Selecting studies with low risk of bias in kidney recipients suggests donor sodium dysregulation is unlikely to affect kidney graft function. Despite the consistency of the results, the certainty in evidence remains low.

We identified only two studies reporting on heart recipient mortality [9, 11]. Kaczmarek, *et al*, found no association between donor sodium levels and recipient mortality, but the division of donor sodium levels into four quartiles may have underestimated the true effect of extreme hypernatremia. Hoefner, *et al*, found extremes of both hyper- and hyponatremia to be associated with a 25% increased risk of mortality (HR: 1.25, 95% CI 1.04–1.50). Donor hemodynamic status was not controlled for, raising concerns for risk of bias in the large effect size they reported. 

The studies investigating the relationship between donor sodium levels and the success of transplanted organs leave us with some conflicting evidence as well. Achieving a serum sodium target of 135–160 mEq/L predicted successful transplantation of four or more organs per donor in the study by Malinoski, *et al*, but Selck, *et al*, did not identify donor sodium as a predictor of the number of organs transplanted. Selck, *et al*, adjusted for confounders such as desmopressin and diuretic administration in their multivariable regression analysis while Malinoski, *et al*, did not. Desmopressin and diuretic administration were associated with increased organ yield, so undetected collinearity could account for the association of donor sodium identified in the latter.

The studies we identified are mixed in their conclusions, and more specifically in their sodium targets, making it difficult to recommend an optimal donor sodium range. Despite this, our review raises a salient point: Perhaps more important than the degree of dysregulation is why the dysregulation is present. The majority of high-quality studies with low risk of bias controlled for donor hemodynamics, volume status, and desmopressin administration, and found no association between dysregulated donor sodium levels and transplant outcomes. Clinical practice guidelines may consider reframing dysregulated donor sodium levels as a symptom of hemodynamic derangement rather than an electrolyte derangement. It is imperative that future studies control for donor volume status, hemodynamics and hormonal therapy when evaluating the clinical effects of donor sodium dysregulation.

In the face of inconclusive evidence regarding the effect of donor sodium levels on transplant outcomes, the very low certainty in evidence may still sway clinical practice guidelines to recommend maintaining donor serum sodium concentration in the normal range (135–145 mEq/L) in potential neurologically deceased donors. There could be a potential benefit to normalizing deranged serum sodium levels (especially, if they are associated with deranged hemodynamics) and there is no evidence of harm from this practice. Neurologically deceased donors require sodium correction prior to death declaration to eliminate confounders, making this practice recommendation a logical sequela of the current standard of care.

Deceased donors with dysregulated donor sodium levels have been considered marginal, a label that risks the discard of a valuable resource [37]. The results of our systematic review challenge this notion: while the studies are collectively heterogenous, the conclusions show some convergence when looking at high quality studies with low risk of bias and stratifying the results based on target organs: donor sodium dysregulation is unlikely to affect kidney graft function, or survival of heart and kidney recipients. We question whether a universal, optimal sodium target for donors even exists, and whether the paradigm of care should shift to a more individualized approach based on the candidacy of organs for donation.

Strengths of our review included a sensitive search strategy with no limitation on publication type. Abstract screening, full-text review, data extraction, and risk of bias assessment were done independently by two authors to minimize bias and selective reporting. We applied GRADE to assess certainty in evidence. However, we are restricted in making strong conclusions in the light of the heterogeneous studies and very low certainty in evidence for all of the outcomes. This review is limited by the sampling method used in the study cohorts we included; the majority of these studies recruited patients from registries of transplant recipients and identified relevant donor factors of transplanted organs. This creates a selection bias against potential donors who were never converted to actual donors, whose phenotype is expected to differ when compared to successful donors. 

In conclusion, the effects of dysregulated donor serum sodium on recipient outcomes are heterogenous across studies. Although based on low certainty in evidence, high quality studies with low risk of bias suggest that dysregulated donor sodium does not independently harm kidney graft function, or increase mortality of heart and kidney recipients. Our review also challenges the notion that donors with dysregulated sodium are “marginal,” underscoring the need for future research to strengthen the certainty in evidence, and inform the optimal sodium target for lung, kidney, heart, and pancreas donors.
